# Transdermal opioid patch in treatment of paroxysmal autonomic instability with dystonia with multiple cerebral insults: A case report

**DOI:** 10.1097/MD.0000000000022536

**Published:** 2020-10-02

**Authors:** Sung-Woon Baik, Dong-Ha Kang, Gi-Wook Kim

**Affiliations:** a Department of Physical Medicine and Rehabilitation, Jeonbuk National University Medical School; b Research Institute of Clinical Medicine of Jeonbuk National University-Biomedical Research Institute of Jeonbuk National University Hospital; c Translational Research and Clinical Trial Center for Medical Device, Jeonbuk National University Hospital, Jeonju, Republic of Korea.

**Keywords:** autonomic nervous system, brain injury, dystonia, opioid

## Abstract

**Rationale:**

Paroxysmal autonomic instability with dystonia (PAID) is an underdiagnosed syndrome that describes a collection of symptoms following diverse cerebral insults, such as traumatic brain injury, hydrocephalus, hemorrhagic stroke, or brain anoxia. It is manifested by systemic high blood pressure, hyperthermia, tachycardia, tachypnea, diaphoresis, intermittent agitation, and certain forms of dystonia.

**Patient concerns:**

A semi-comatose 46-year-old man was transferred from the regional rehabilitation hospital with various complaints involving fluctuating vital signs, including uncontrolled hyperthermia, hypertension, tachycardia, and tachypnea, and dystonia in all extremities. The patient underwent brain surgery for astrocytoma in 1996. The patient also had a history of first ischemic stroke on the basal ganglia in 2008 and a second one in the same area in 2017.

**Diagnosis:**

The laboratory, electrocardiography, and radiologic findings were normal. Brain imaging indicated an old infarction on the basal ganglia with hydrocephalus. Tractography using diffusion tensor imaging showed discontinuity of multiple tracts, and electrophysiologic tests, such as evoked potentials, displayed an absent response. Based on the dysautonomic symptoms and brain evaluations, the physiatrist diagnosed the patient with PAID.

**Interventions:**

Bromocriptine, propranolol, and clonazepam were administered sequentially, but autonomic instability persisted. Then, intravenous opioid was administered, and fluctuations in body temperature, heart rate, and respiratory rate, as well as decerebrate-type dystonia were improved. However, simultaneously, drug-induced severe hypotension developed (systolic blood pressure, 57 mm Hg). Subsequently, a transdermal opioid (fentanyl) patch for PAID was applied once every 3 days.

**Outcomes:**

Ultimately, all vital signs and dystonia were managed without further complications, and the patient was discharged.

**Lessons:**

A patient diagnosed with PAID following multiple cerebral insults was observed, whose condition was controlled by application of opioid patch rather than by intravenous or oral routes. A transdermal opioid patch, such as fentanyl patch, can thus be effective in the treatment of patients with PAID following multiple cerebral insults.

## 1 Introduction

Paroxysmal autonomic instability with dystonia (PAID) has been known as an underdiagnosed syndrome following diverse cerebral insults, which is manifested by episodic hypertension, hyperthermia, tachycardia, tachypnea, diaphoresis, agitation, hypertonia, and extensor posturing.[^1^,^2^] According to a previous study on this sympathetic hyperactivity, the incidence ranges from 8% to 33%, depending on underlying brain damage: traumatic brain injury (79.4%), hypoxic brain injury (9.7%), cerebrovascular disorder (5.4%), hydrocephalus (2.6%), tumor (0.6%), and infectious brain injury (0.3%).[^1^,^2^,^3^,^4^,^5^,^6^,^7^,^8^] The mechanism of PAID has been known to be disconnection of the cortical inhibitory center or imbalance between brain excitation and inhibition.[^9^,^10^] To prove these mechanisms and the underlying brain pathology, brain diffusion tensor imaging (DTI), which can show the disconnection of cortical, subcortical, and stem lesions, reportedly plays an effective role.^[10]^ Several treatments have been introduced to ameliorate the sympathetic hyperactivity, including opioids, intravenous (IV) anesthetics (propofol), β-blockers (propranolol, labetalol, metoprolol), α2-agonists (oral clonidine and IV dexmedetomidine), and others (bromocriptine, benzodiazepines).[^1^,^2^,^11^] However, these medications have significant side effects, notably respiratory depression, sedation, bradycardia, and hypotension, which can cause life-threatening attacks on patients with PAID after severe brain injury.^[11]^

In this study, we report a severe and unique case of a patient with PAID following multiple cerebral insults due to brain tumor and cerebral infarction, most notably hypotensive shock after IV morphine infusion. Treatment with a fentanyl patch can be an effective therapeutic option for patients with PAID who have uncontrolled sympathetic hyperactivity and severe side effects of IV morphine.

## 2 Case Presentation

A 46-year-old man with a history of intermittent high fever for 9 days was transferred to the emergency department because of uncontrolled fever with temperatures as high as 39°C to 40.6°C, accompanying hypertension (systolic blood pressure [BP], 168–190 mm Hg), tachycardia (133–155/min), tachypnea (35–48/min), and dystonia in all extremities (decerebrate posture). The patient underwent brain surgery for astrocytoma in 1996 and had ischemic stroke at the right basal ganglia twice in 2008 and 2017 before the current visit. He had been admitted to the regional rehabilitation hospital and at that time had been in a semi-comatose state and had double spastic hemiplegia based on Modified Ashworth Scale 1 ± 2. At the initial admission to our hospital, he was in a semi-comatose state with aggravated spasticity (Modified Ashworth Scale 3–4). Upon admission to the regional rehabilitation center, the patient received levetiracetam 500 mg TID, tizanidine hydrochloride 1 mg TID, and baclofen 10 mg 1T BID. Although the laboratory test and chest radiography findings at the local rehabilitation center were normal, intermittent episodes of high fever were continuous, despite treatment with acetaminophen and a nonsteroidal anti-inflammatory drug.

We evaluated the laboratory test, cerebral fluid (CSF) analysis, chest and abdomen radiography, and brain magnetic resonance imaging (MRI) for differential diagnosis of systemic infection or inflammation, especially infection of the central nervous system or recurrence of stroke. In the laboratory test, the patient had a white blood cell count of 13,120/mL (reference range, 4500–11,000), but other inflammatory and infectious markers, such as erythrocyte sedimentation rate and C-reaction protein level, were normal. Lumbar puncture was performed, and the results of the CSF analysis were normal. Blood, urine, and CSF specimens were sent for culture study, which showed no growth. Chest and abdomen radiography findings were normal. The results of brain MRI and electroencephalography showed no newly detected lesions. The infectious disease specialist did not recommend antibiotics, in view of the limited evidence of underlying infectious and inflammatory disease. Although the cardiologist performed transthoracic echocardiography to detect anatomical and functional cardiac diseases causing tachycardia, high BP, and its related complications, the results showed normal systolic function and ejection fraction of 60% with no other heart disorders.

**Figure 1:**
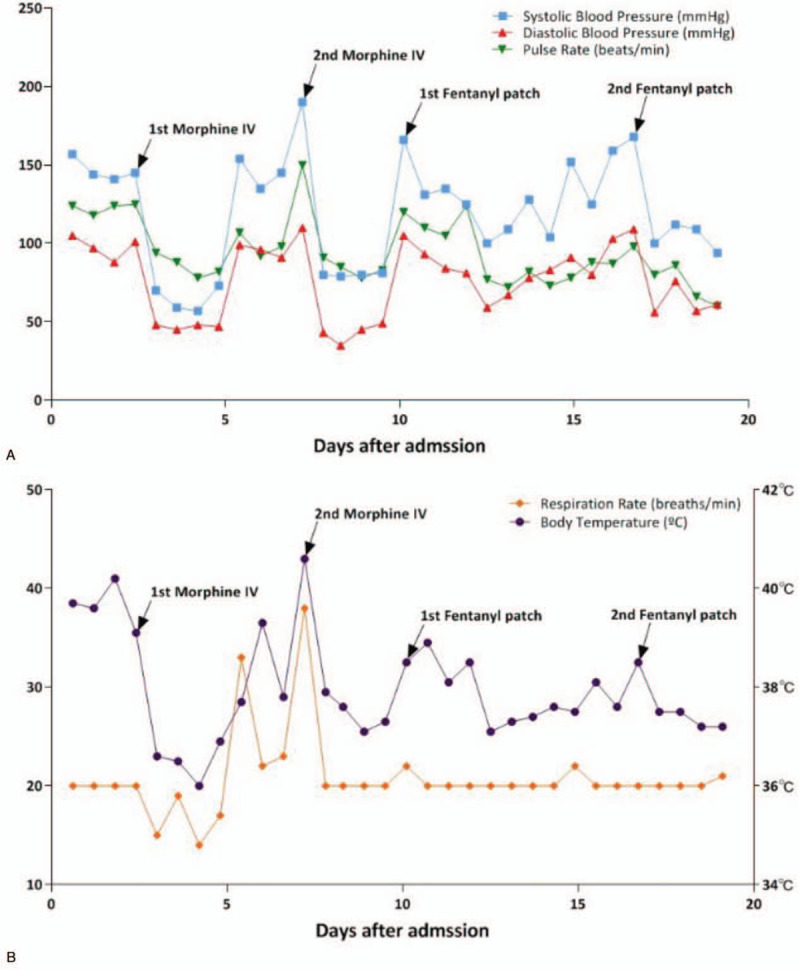
Change in vital signs during treatment in patient with PAID. (A) Blood pressure and pulse. (B) Body temperature and respiratory rate. Vital signs of the patient with PAID in the emergency room were unstable. After intravenous morphine administration, vital signs other than blood pressure and dystonic movements were managed effectively. However, systolic blood pressures remained below 90 mm Hg. After application of a transdermal opioid patch (fentanyl patch), vital signs and dystonic movements were managed without further complications. PAID = paroxysmal autonomic instability with dystonia.

Informed consent was obtained from the patient's mother before treatment. Based on previous reports of effective therapeutic options for patients with PAID, we administered bromocriptine 1.435 mg BID, propranolol hydrochloride 4 mg BID, and clonazepam 0.25 mg BID. However, intermittent fever with temperature as high as 39.0°C continued, and the patient maintained decerebrate posturing. Intravenous morphine at 1 mg/h was administered on day 2, after which systolic BP decreased drastically to 57 mm Hg, with low heart rate, temperature, and respiratory rate (Fig. 1). The patient was hydrated with crystalloid fluids and transferred to the intensive care unit for close observation of low BP. In the days following the patient's transfer to the intensive care unit, he developed high fever and hypertension with other sympathetic hyperactivity symptoms. Despite treatment with 0.5 mg of IV morphine, hypotensive events continued to reoccur, combined with bradycardia and hypothermia.

**Figure 2:**
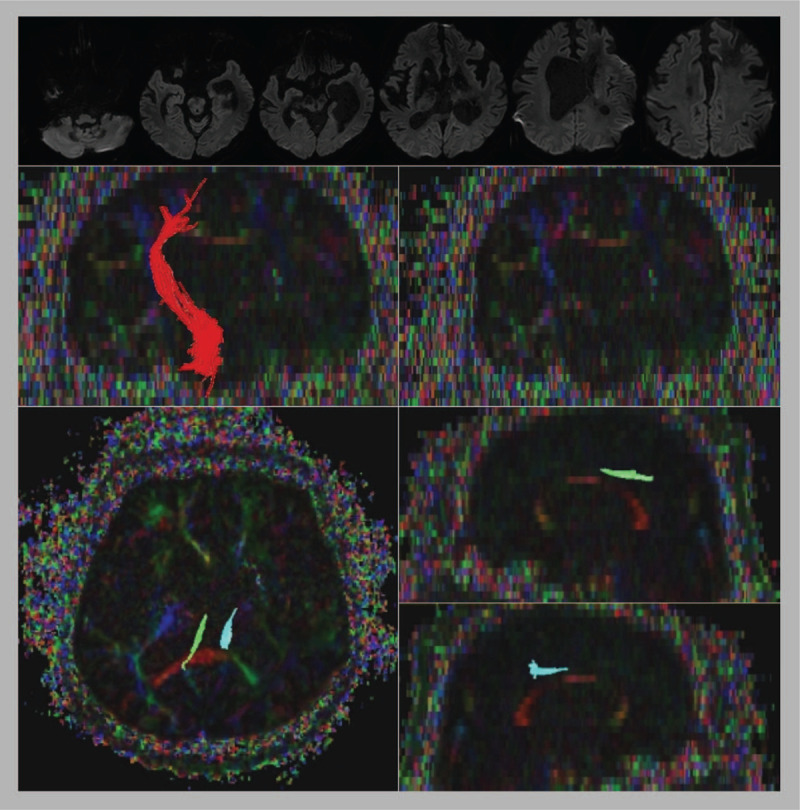
Brain magnetic resonance images and evoked potential studies in patient with PAID. (A) At initial presentation, brain diffusion MRI showed no acute lesions. (B) Tractography of the corticospinal tract was observed only on the left side. In addition, the spinothalamic tract was not detected (C), and disrupted white matters were observed in the cingulum (D–F). The motor evoked potential study, stimulating each hemisphere (upper line, right hemisphere; lower line, left hemisphere) and recording at the first digit interosseous (G) and tibialis anterior muscle (H), showed mild delayed latency and decreased amplitude in the right hemisphere.

On the fifth hospital day, the patient was administered a 25 mg (transdermal) fentanyl patch. During this period, he no longer showed life-threatening hypotension, but a decrease in fluctuating episodes of hypertension, tachycardia, hyperthermia, and tachypnea with dystonia. In the follow-up laboratory test, white blood cell counts were normalized to 9240/mL, and other results were normal. For further evaluation of cortical and subcortical dissociation, brain DTI was performed, and tractography showed discontinuity of multiple tracts (Fig. 2). On electrophysiologic tests, somatosensory and motor evoked potentials from the contralateral hemisphere showed delayed latency and decreased amplitude in the right hemisphere.

After 3 days of treatment with fentanyl patch, which had by then lost its therapeutic effect, increased BP, heart rate, temperature, and respiratory rate and dystonia recurred. Therefore, the patient's transdermal fentanyl patch was replaced every 3 days, as a result of which the vital signs remained stable, while dystonia improved without further complications. The patient's unstable vital signs successfully improved over 2 months of using the transdermal fentanyl patch, after which the patient was lost to follow-up.

## 3 Discussion

PAID mostly presents in patients with a single severe brain injury due to trauma or hypoxic events, occurring within 1 month after the underlying brain injury.[^3^,^4^,^5^,^6^,^7^,^8^,^13^] In our study, the patient's PAID occurred 3 months after the most recent ischemic stroke and coincided with severe injury of the brain astrocytoma and 2 occurrences of ischemic stroke. β-blockers (propranolol), bromocriptine, and clonazepam were administered to the patient, but the sympathetic storms continued. Treatment with IV morphine decreased the BP, pulse rate, respiratory rate, and temperature and improved dystonia, but, at the same time, the patient developed life-threatening hypotension, with bradycardia, hypothermia, and bradypnea. However, after treatment with a transdermal fentanyl patch, the patient's intermittent episodes of sympathetic hyperactivity were ameliorated without further complications.

In previous studies, the pathophysiology of PAID involved disconnecting the cortical inhibitory center, such as the insular and cingulate gyri, from the hypothalamic, diencephalic, and brainstem centers, which control sympathetic activity.[^9^,^10^] In addition to the disconnection theory, the excitation and inhibition ratio model has been introduced, which suggests dysfunction of the efferent inhibitory pathway and afferent excitatory pathway.^[9]^ DTI plays an important role in the understanding of these neuroanatomical and associated impairments.^[10]^ In one previous study, tractography analysis using DTI in 102 patients with severe traumatic brain injury with paroxysmal autonomic hyperactivity showed a significant disconnection in the posterior corpus callosum and posterior limb of the internal capsule.^[10]^ In our study, brain diffusion MRI did not show acute detected lesions, but the patient exhibited absence of both the right corticospinal and spinothalamic tracts, accompanied by disconnection of the corpus callosum in tractography using brain DTI. Additionally, our electrophysiologic study showed delayed latency and decreased amplitude in the right hemisphere. Unlike single severe brain injury, multiple brain lesions in this patient might have progressively worsened the disconnection of the cerebral autonomic controlling system, although clinical manifestations and brain MRI have limitations for understanding both the progression and severity of these diseases. DTI and electrophysiologic study can provide further information on this.

Therapeutic approaches to PAID involve removing the noxious stimulus that has provoked the symptoms, decreasing the hyperactive autonomic systems, and minimizing the effect on other organs through conservative treatment.[^1^,^2^,^11^] β-adrenergic drugs are used to target tachycardia, hypertension, diaphoresis, and dystonia by reducing adrenergic responsiveness, which can cause bradycardia, hypotension, arrhythmia, sleep disturbance, and masked hypoglycemia.[^1^,^2^,^11^,^12^] Neuromodulators, such as bromocriptine, gabapentin, and baclofen, have been suggested as an additional treatment for fever, diaphoresis, spasticity, and dystonia.[^1^,^2^,^11^,^12^] Opioids, particularly IV morphine, have been used as an initial treatment in PAID and act through central and peripheral suppression of opioid receptors, targeting hypothalamic regulation.^[12]^ Opioid therapy is used mainly for treating hypertension, allodynia, and tachycardia, but these therapeutic effects may overcontrol BP or respiration, causing life-threatening conditions, such as respiratory depression or hypotension.^[12]^ Since patients with PAID have severe brain injuries in the acute or subacute phase, they are more susceptible to these side effects. In the present case, the patient's sympathetic hyperactivity (high BP, tachycardia, tachypnea, and fever) dramatically improved by IV morphine, but this improvement coincided with drastic hypotension with bradycardia and low temperature. In previous case reports, 2 patients with a large tumor in their brainstem that was encroaching on the hypothalamus had PAID syndrome.^[13]^ β-blockers, diazepam, neuromodulators, such as gabapentin, and baclofen were administered, but did not improve sympathetic hyperactivity.^[13]^ However, management by a transdermal fentanyl patch progressively ameliorated patients’ sympathetic symptoms.^[13]^ Constipation was the only minor complication in these cases, and no other major side effects occurred requiring termination of the treatment.^[13]^ In the present case, when treated with a transdermal fentanyl patch the patient also showed a decrease in all sympathetic symptoms, without severe complications due to over-controlling (hypotension). Significantly, when the fentanyl patch needed to be removed and reapplied 3 days after the initial application, hypertension, tachycardia, tachypnea, and fever all recurred, while reapplying the patch again relieved these symptoms. These consistent responses confirm the effectiveness of treatment by fentanyl patch. Based on both the present case and the 2 previous cases, the transdermal fentanyl patch was relatively progressive and safe in its therapeutic effects, while it also improved sympathetic hyperactivity even though this had proved resistant to other medications, such as β-blockers, benzodiazepam, neuromodulator, and antispasmodic medication.

In our study, a patient with PAID, diagnosed with severe disconnection of the cortex, subcortex, and corpus callosum via brain DTI, was successfully treated for sympathetic hyperactivity with a transdermal fentanyl patch and without incurring additional complications, such as opioid-induced hypotension. For differential diagnosis of acute brain lesion and its related seizure disorders, brain diffusion MRI and electroencephalography performed on the patient revealed no new lesions. Additionally, we evaluated brain DTI and performed a motor and somatosensory-evoked potential study to evaluate the severity of the cortical, subcortical, and brainstem disconnection that caused PAID. DTI of the patient showed the absence of the right corticospinal and spinothalamic tracts and discontinuation of the corpus callosum, consistent with that of a previous study.^[10]^ The initial opioid therapy with IV morphine caused life-threatening hypotension with bradycardia, bradypnea, and hypothermia, while the second trial with a half dose of IV morphine also produced the same side effects, without bradypnea. Therefore, the transdermal fentanyl patch can be considered both a safe and effective therapeutic option for the treatment of PAID.

## Author contributions

**Conceptualization, formal analysis, and writing – review & editing:** Gi-Wook Kim

**Data curation and investigation:** Dong-Ha Kang

**Writing – original draft:** Sung-Woon baik
